# Development and validation of a novel online calculator for estimating survival benefit of adjuvant transcatheter arterial chemoembolization in patients undergoing surgery for hepatocellular carcinoma

**DOI:** 10.1186/s13045-021-01180-5

**Published:** 2021-10-12

**Authors:** Lei Liang, Chao Li, Ming-Da Wang, Hong Wang, Ya-Hao Zhou, Yong-Yi Zeng, Wan-Guang Zhang, Ting-Hao Chen, Nan-Ya Wang, Jie Li, Yao-Ming Zhang, Yu Wang, Wei-Min Gu, Hao Xing, Yong-Kang Diao, Wan Yee Lau, Cheng-Wu Zhang, Timothy M. Pawlik, Feng Shen, Dong-Sheng Huang, Tian Yang

**Affiliations:** 1 Department of Hepatobiliary, Pancreatic and Minimal Invasive Surgery, Zhejiang Provincial People’s Hospital, People’ Hospital of Hangzhou Medical College, No. 158, Shangtang Road, Hangzhou, 310014 Zhejiang China; 2 Key Laboratory of Tumor Molecular Diagnosis and Individualized Medicine of Zhejiang Province, Hangzhou, China; 3 Department of Hepatobiliary Surgery, Eastern Hepatobiliary Surgery Hospital, Second Military Medical University (Navy Medical University), Shanghai, China; 4 Department of General Surgery, Liuyang People’s Hospital, Hunan, China; 5 Department of Hepatobiliary Surgery, Pu’er People’s Hospital, Yunnan, China; 6 Department of Hepatobiliary Surgery, Mengchao Hepatobiliary Hospital, Fujian Medical University, Fujian, China; 7 Department of Hepatic Surgery, Tongji Hospital, Huazhong University of Science and Technology, Wuhan, China; 8 Department of General Surgery, Ziyang First People’s Hospital, Sichuan, China; 9 The Cancer Center, the First Hospital of Jilin University, Jilin, China; 10 Department of Hepatobiliary Surgery, Fuyang People’s Hospital, Anhui, China; 11 The Second Department of Hepatobiliary Surgery, Meizhou People’s Hospital, Guangdong, China; 12 Department of Hepatobiliary Surgery, Chongqing University Cancer Hospital, Chongqing, China; 13 The First Department of General Surgery, The Fourth Hospital of Harbin, Heilongjiang, China; 14 Faculty of Medicine, The Chinese University of Hong Kong, Shatin, New Territories, Hong Kong China; 15 Department of Surgery, Ohio State University, Wexner Medical Center, Columbus, OH USA; 16 School of Clinical Medicine, Hangzhou Medical College, Hangzhou, Zhejiang China

**Keywords:** Hepatocellular carcinoma, Hepatectomy, Transcatheter arterial chemoembolization, Adjuvant therapy, Survival

## Abstract

**Background and aims:**

Although adjuvant transcatheter arterial chemoembolization (TACE) for resected hepatocellular carcinoma (HCC) may improve survival for some patients, identifying which patients can benefit remains challenging. The present study aimed to construct a survival prediction calculator for individualized estimating the net survival benefit of adjuvant TACE for patients with resected HCC.

**Methods:**

From a multicenter database, consecutive patients undergoing curative resection for HCC were enrolled and divided into the developing and validation cohorts. Using the independent survival predictors in the developing cohort, two nomogram models were constructed for patients with and without adjuvant TACE, respectively, which predictive performance was validated internally and externally by measuring concordance index (C-index) and calibration. The difference between two estimates of the prediction models was the expected survival benefit of adjuvant TACE.

**Results:**

A total of 2514 patients met the inclusion criteria for the study. The nomogram prediction models for patients with and without adjuvant TACE were, respectively, built by incorporating the same eight independent survival predictors, including portal hypertension, Child–Pugh score, alpha-fetoprotein level, tumor size and number, macrovascular and microvascular invasion, and resection margin. These two prediction models demonstrated good calibration and discrimination, with all the C-indexes of greater than 0.75 in the developing and validation cohorts. A browser-based calculator was generated for individualized estimating the net survival benefit of adjuvant TACE.

**Conclusions:**

Based on large-scale real-world data, an easy-to-use online calculator can be adopted as a decision aid to predict which patients with resected HCC can benefit from adjuvant TACE.

**Supplementary Information:**

The online version contains supplementary material available at 10.1186/s13045-021-01180-5.

To the editor,

Hepatocellular carcinoma (HCC) is the third most common cause of cancer-related mortality worldwide [1]. Surgical resection represents a common approach to treat HCC and provides the possibility of cure [2]. Long-term prognosis after HCC resection is, however, still poor due to the high incidence of recurrence [3–5]. Transcatheter arterial chemoembolization (TACE) has been used in the postoperative setting as a means to decrease risk of recurrence and improve survival [6–8]. Whereas, in clinical practice, controversy persists relative to the role of adjuvant TACE for resected HCC [9–11]. The reasons for these disparate results are undoubtedly multifactorial, yet may relate to patient selection. Specifically, only certain high-risk patients with resected HCC may benefit from adjuvant TACE [12]. The objective of the current study was to construct a decision aid using a large multicenter database to predict which patient with resected HCC had a survival benefit from adjuvant TACE. In addition, we sought to estimate the magnitude of the survival benefit for given individual patients. A web-based decision tool was provided for clinicians and patients to aid in the decision-making process regarding adjuvant TACE after HCC resection. Patients and methods for this study are described in detail in Additional file 1.

## Overall survival

All 2514 patients with HCC underwent curative liver resection were included (Additional file 2: Figure S1). Among them, 1755 and 759 patients were randomly segregated to the development and validation cohort, respectively (Table 1). Compared with patients who did not receive adjuvant TACE, patients who had adjuvant TACE had a longer survival in both the development and validation cohorts (all *P* < 0.001) (Additional file 3: Figure S2).

**Table 1:** Baseline characteristics of patients with and without adjuvant TACE in the developing and validation cohorts

Variables	The developing cohort (N = 1755)		The validation cohort (N = 759)	
	With adjuvant TACE (n = 533)	Without adjuvant TACE (n = 1222)	With adjuvant TACE (n = 224)	Without adjuvant TACE (n = 535)
*Preoperative variables*				
Male sex	476 (89.3)	1080 (88.4)	198 (88.4)	468 (87.5)
Age > 65 years	89 (16.7)	260 (21.3)	29 (12.9)	115 (21.5)
Co-morbid illness	111 (20.9)	228 (18.7)	72 (32.1)	160 (29.9)
PS, 1–2/0	161/372 (30.2/69.8)	345/877 (28.2/71.8)	35/189 (15.7/84.3)	82/453 (15.3/84.7)
ASA score > 2	56 (10.5)	182 (14.9)	19 (8.5)	81 (15.1)
Etiology of liver disease, HBV/HCV/HBV + HCV/other	446/26/15/46 (83.7/4.9/2.8/8.6)	1010/73/27/112 (82.7/6.0/2.2/9.2)	193/9/4/18 (86.2/4.0/1.8/8.0)	446/32/9/48 (83.4/6.0/1.7/9.0)
Cirrhosis	391 (73.4)	958 (78.4)	157 (70.1)	421 (78.7)
Portal hypertension	115 (21.6)	329 (26.9)	37 (16.5)	129 (24.1)
Child–Pugh grade, A/B	489/44 (91.7/8.3)	1075/147 (88.0)	207/17 (92.4/7.6)	467/68 (87.3/12.7)
Preoperative ALT level > 40 U/L	277 (52.0)	683 (55.9)	111 (49.6)	289 (54.0)
Preoperative AST level > 40 U/L	284 (53.3)	703 (57.5)	141 (62.9)	314 (58.7)
Preoperative AFP level > 400 ug/L	206 (38.6)	489 (40.0)	87 (38.8)	198 (37.0)
Maximum tumor size, ≥ 10.0/5.0–9.9/< 5.0 cm	128/212/193 (24.0/39.8/36.2)	204/443/575 (16.7/36.3/47.1)	45/96/83 (20.1/42.9/37.1)	77/200/258 (14.4/37.4/48.2)
Tumor number, ≥ 3/2/1	91/82/360 (17.1/15.4/67.5)	159/121/942 (13.0/9.9/77.1)	33/35/156 (14.7/15.6/69.6)	70/50/415 (13.1/9.3/77.6)
Macrovascular invasion	67 (12.6)	104 (8.5)	35 (15.6)	50 (9.3)
*Intraoperative variables*				
Intraoperative blood loss > 600 mL	117 (22.0)	263 (21.5)	42 (18.8)	104 (19.4)
Intraoperative blood transfusion	145 (27.2)	307 (25.1)	45 (20.1)	132 (24.7)
Operation time > 180 min	86 (16.1)	214 (17.5)	30 (13.4)	84 (15.7)
Anatomical resection	145 (27.2)	331 (27.1)	63 (28.1)	129 (24.1)
Major hepatectomy	157 (29.5)	318 (26.0)	56 (25.0)	132 (24.7)
*Postoperative pathological variables*				
Microvascular invasion	306 (57.4)	619 (50.7)	134 (59.8)	270 (50.5)
Poor tumor differentiation	397 (74.5)	918 (75.1)	175 (78.1)	415 (77.6)
Incomplete tumor encapsulation	334 (62.7)	785 (64.2)	155 (69.2)	344 (64.3)
Resection margin < 1 cm	179 (33.6)	386 (31.6)	76 (33.9)	162 (30.3)

*AFP* alpha-fetoprotein, *ALT* alanine aminotransferase, *ASA* American Society of Anesthesiologists, *AST* aspartate transaminase, *HBV* hepatitis B virus, *HCV* hepatitis C virus, *PS* performance status, *TACE* transcatheter arterial chemoembolization

## Independent predictors of survival

Univariable and multivariable Cox regression analyses of the development cohort demonstrated that independent predictors associated with overall survival after HCC resection among patients treated with and without adjuvant TACE included portal hypertension, Child–Pugh grade, preoperative AFP level, tumor size, tumor number, macrovascular invasion, microvascular invasion, and resection margin (all *P* < 0.05). (Additional file 4: Table S1 and Additional file 5: Table S2).

## Development of the prediction models

**Fig. 1:**
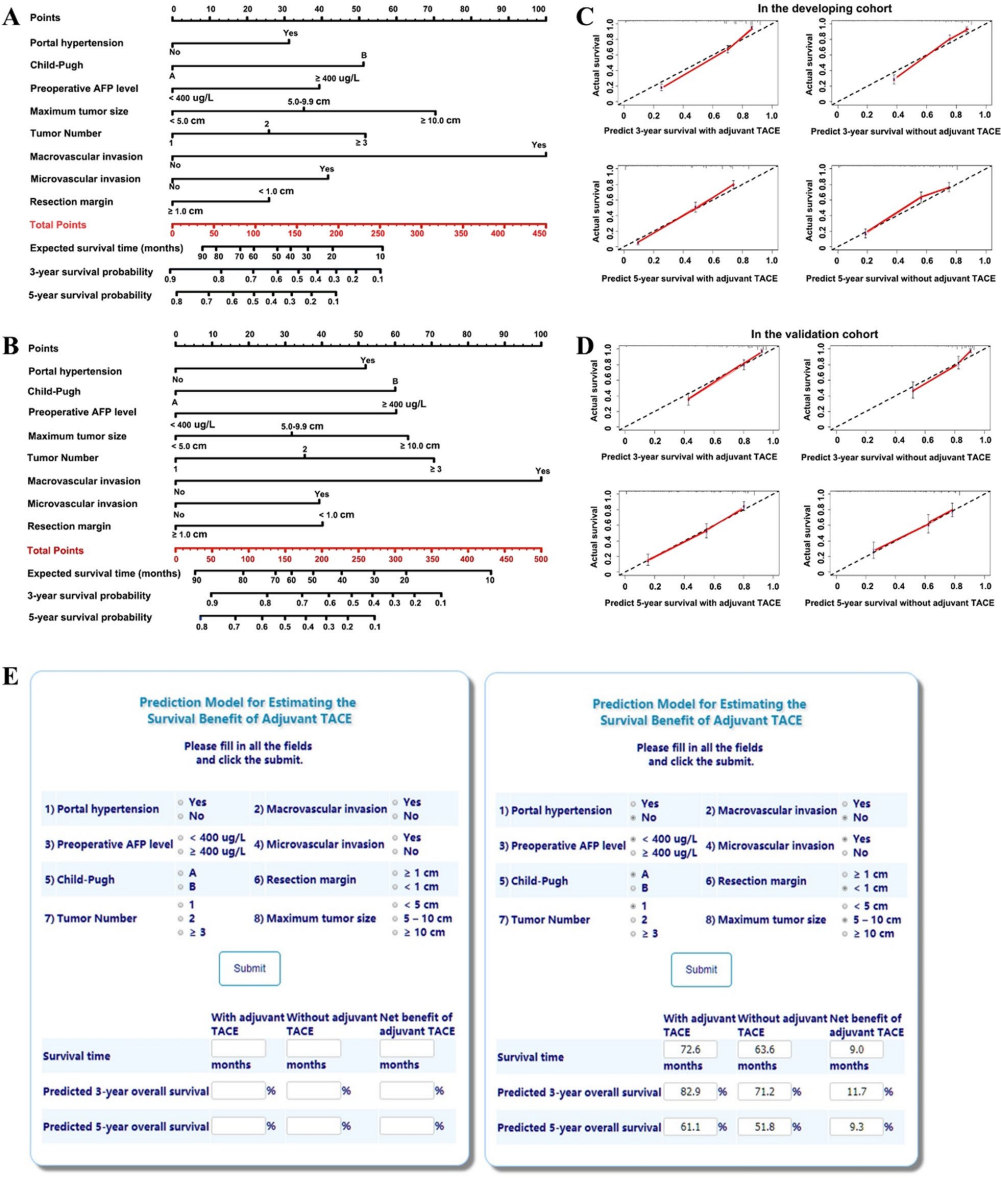
Nomograms to calculate the expected survival time, and 3- and 5-year survival probabilities for **a** patients who attempt to undergo adjuvant TACE and **b** patients who attempt not to undergo adjuvant TACE. Thus, the difference of the expected values between the two estimates is the expected net survival benefit of adjuvant TACE. Calibration plots of the models for predicting 3-year and 5-year survival for patients with and without adjuvant TACE **c** in the developing cohort and **d** in the validation cohort, respectively. **e** Screenshots of the web-based calculator for individualized estimates of the expected net survival benefit of adjuvant TACE for patients with resected hepatocellular carcinoma. Website: http://www.asapcalculate.top/Cal5_en.html. For example, suppose there is a male patient without portal hypertension (Child–Pugh A) who have underwent curative resection for a single HCC tumor (tumor size: 8.0 cm, without macrovascular invasion but with microvascular invasion). His preoperative AFP level was 718 ug/L (≥ 400 ug/L), and the tumor resection margin was 0.8 cm (< 1 cm). After putting these data into these specific parameters, we can get the expected net survival time benefit of adjuvant TACE was 9.0 months, and the net survival benefits of 3-year and 5-year survival rates are 11.7% and 9.3%, respectively

Two different nomogram models that integrated independent factors associated with overall survival were constructed to predict outcomes among patients who did and did not receive adjuvant TACE (Fig. 1a, b). To estimate the net survival benefit from adjuvant TACE, these two nomograms were compared and the difference between the two estimates was the expected net survival benefit from the addition of adjuvant TACE.

## Validation of the prediction models

Bootstrapping with 400 resamples in the development cohort demonstrated good predictive performance, with the C-indexes of 0.791 (95% CI 0.742–0.840) and 0.810 (95% CI 0.779–0.841) for patients with and without adjuvant TACE, respectively. Accordingly, the C-indices were 0.756 (95% CI 0.678–0.834) and 0.765 (95% CI 0.720–0.810) in the validation cohort. There was also good calibration curve to predict 3- and 5-year survival probabilities among patients treated with and without adjuvant TACE, respectively (all *P* > 0.05) (Fig. 1c, d).

## Construction of the online calculator

Based on the formula of the nomogram prediction models, an Internet browser-based software tool was constructed to predict the net survival benefit of adjuvant TACE for an individual patient, including the expected net survival time, and the increased 3- and 5-year survival probabilities (Fig. 1e). The corresponding score and the formula to calculate survival probability were provided (Additional file 6: Table S3). The online calculator is available for free use at: http://asapcalculate.top/Cal5_en.html. After the user inputs all the requested information relative to the prognostic factors, the predicted survival improvement associated with the addition of adjuvant TACE, including the expected survival time and the 3- and 5-year survival probabilities, is generated and displayed.

In summary, a survival prediction model that incorporated eight independent variables associated with survival was constructed to derive an individualized estimate of the net survival benefit of adding adjuvant TACE to a patient’s post-resection HCC treatment plan. The nomograms and online calculator had good predictive accuracy, and discrimination was validated. The calculator may help provide an estimate of the net survival benefit associated with adjuvant TACE for an individual patient following HCC resection. This tool may assist clinicians and patients in quantifying the benefit of adjuvant TACE after HCC resection and inform real-word discussions on this topic.

## Supplementary Information

**Additional file 1.** Patients and methods.

**Additional file 2: Figure S1.** Flow chart of patient inclusion.

**Additional file 2: Figure S2.** (A) Kaplan-Meier curves of overall survival between patients in the development and validation cohorts (log-rank test, P = 0.594); (B) Kaplan-Meier curves of overall survival between patients with and without adjuvant TACE in the developing cohort (log-rank test, *P* < 0.001); and (C) Kaplan-Meier curves of overall survival between patients with and without adjuvant TACE in the validation cohort (log-rank test, *P* < 0.001). Number of patients at risk and censoring were list in the box at the bottom of each plot.

**Additional file 3: Table S1.** Univariable and multivariable Cox-regression analyses of predicting overall survival for patients with resected hepatocellular carcinoma who underwent adjuvant TACE in the developing cohort.

**Additional file 2: Table S2.** Univariable and multivariable Cox-regression analyses of predicting overall survival for patients with resected hepatocellular carcinoma who did not undergo adjuvant TACE in the developing cohort.

**Additional file 2: Table S3.** The corresponding score and the formula of our nomogram models.

## Abbreviations

HCC: Hepatocellular carcinoma
TACE: Transcatheter arterial chemoembolization
RCT: Randomized controlled trial
CT: Computed tomography
MRI: Magnetic resonance imaging
PS: Performance status
ECOG: Eastern cooperativse oncology group
ASA: American society of anesthesiologists
HBV: Hepatitis B virus; HCV, hepatitis C virus
ALT: Alanine aminotransferase
AST: Aspartate transaminase
AFP: Alpha-fetoprotein
C-index: Concordance index
CI: Confidence interval
HR: Hazard ratio

## Declarations

### Congress publication

The abstract of this study has been presented as an oral presentation in the Congress of “the 11th Asia-Pacific Primary Liver Cancer Expert, APPLE 2021” (August 13–15, 2021, Korea), and has been also presented as an oral presentation in the Congress of “the LIVER WEEK 2021” (May 13–15, 2021, Korea) and won a “Foreign Investigator Award”.
