# Supplementary material for: Development and validation of a novel online calculator for estimating survival benefit of adjuvant transcatheter arterial chemoembolization in patients undergoing surgery for hepatocellular carcinoma
Source: J Hematol Oncol. 2021 Oct 12;14:165. doi: 10.1186/s13045-021-01180-5 (PMC8507320; doi:10.1186/s13045-021-01180-5)
Supplement: Supplementary file 1 — Additional file 1. Patients and methods. [file 13045_2021_1180_MOESM1_ESM.docx]

**Patients and Methods**

***Study Population***

Data on consecutive patients who underwent curative-intent liver resection for HCC at 12 Chinese hospitals from January 2006 to December 2017 were collected (Zhejiang Provincial People's Hospital, Eastern Hepatobiliary Surgery Hospital, Liuyang People’s Hospital, Pu’er People’s Hospital, Mengchao Hepatobiliary Hospital, Tongji Hospital, Ziyang First People’s Hospital, the First Hospital of Jilin University, Fuyang People’s Hospital, Meizhou People's Hospital, Chongqing University Cancer Hospital, the Fourth Hospital of Harbin). The clinical diagnosis of HCC was histologically confirmed in each case. Curative resection was defined as complete resection of all tumor nodules with a microscopic free margin (R0 resection). Patients were excluded who 1) were less than 18 years old; 2) had recurrent HCC; 3) received neoadjuvant or other adjuvant therapies, including radiotherapy and systemic therapy; 4) died within 3 months after surgery; 5) had residual tumor(s) in the liver or extrahepatic metastasis detected by angiography, contrast-enhanced computer tomography (CT) or magnetic resonance imaging (MRI) during follow-up within 3 months after surgery; 6) had incomplete medical records on relevant clinical variables. Using random assignment, 70% of enrolled patients were assigned to the development cohort, while the remaining 30% were assigned to the validation cohort. The study was performed in accordance with the Declaration of Helsinki and the Ethical Guidelines for Clinical Studies in all participating hospitals.

***Follow‑up***

Adjuvant TACE was performed within 4~8 weeks after surgery at all the participating hospitals. All patients were followed-up at each participating hospital after hospital discharge. Postoperative surveillance included physical examination, serum AFP level, ultrasonography or contrast-enhanced CT scan or MRI of the chest and abdomen at least once every two months in the first 6 months after liver resection, and then every three months in the following 18 months, and at 6-monthly intervals thereafter. CT, MRI, angiography, bone scan, or positron emission tomography were performed earlier when recurrence or distant metastasis was suspected. Tumor recurrence was defined as new appearance of an intra- or extrahepatic tumor nodule. Based on the pattern of recurrent disease, residual hepatic functional reserve, and general condition of the patient, further treatment for the recurrent tumor was undertaken at the treating surgeon’s discretion. The treatment decision for recurrent tumor was based on the pattern of recurrent disease, residual hepatic functional reserve, and general condition of the patient. The options included re-resection, TACE, local ablation, radiotherapy, systemic therapy with oral sorafenib, or best supportive treatment, either alone or in combination.

***Study Endpoint and Statistical Analysis***

The endpoint of interest was overall survival, which was calculated from the date of surgery to either the date of death or the date of last follow-up visit. Variables potentially associated with overall survival were selected based on clinical experience and empiric analyses of the data, including sex, age, comorbid illnesses (consists of diabetes mellitus, chronic obstructive pulmonary disease, cardiovascular disease, and renal dysfunction history), performance status (PS) determined by Eastern Cooperative Oncology Group (ECOG), American Society of Anesthesiologists (ASA) score, etiology of liver disease [hepatitis B virus (HBV), hepatitis C virus (HCV), or others], cirrhosis, portal hypertension, Child-Pugh grade, preoperative alanine aminotransferase (ALT), [aspartate](javascript:;) [transaminase](javascript:;) (AST) and alpha-fetoprotein (AFP) level, maximum tumor size, tumor number, macrovascular and microvascular invasion, tumor differentiation, tumor encapsulation, intraoperative blood loss, intraoperative blood transfusion, operation time, resection type (anatomical or non-anatomical), extent hepatectomy (minor or major), and resection margin (< 1 cm or ≥ 1 cm). Portal hypertension was defined by the presence of either esophageal varices or splenomegaly with a decreased platelet count (≤ 100×10^9^/L). Major hepatectomy was defined as resection of three or more Couinaud segments, while minor hepatectomy was resection of fewer than 3 Couinaud segments.

Continuous variables were divided into binary or tertile categories based on previous studies. Categorical variables were expressed as number (proportion, %) and compared using the χ^2^ test or the Fisher’s exact test. Kaplan-Meier curves of cumulative survival were compared using the log rank test. Univariable and multivariable Cox-regression analyses were performed on data in the development cohort to identify independent predictors associated with long-term survival after surgical resection of HCC with versus without adjuvant TACE. Those variables with an univariable P < 0.1 were entered into the multivariable Cox-regression model using a forward stepwise variable selection. These independent predictors were then used to construct two nomogram models to predict three survival-related endpoints, including the expected survival time, and 3- and 5-year survival probabilities. To identify collinearity among variables, all covariates were with a P > 0.05 in the verification of proportional risk hypothesis in each cohort. Interactions among variables were also identified by loglikelihood ratio test. Performance of the two nomogram models was evaluated and validated by concordance index (C-index) and calibration with 400 bootstrap samples, respectively. The C-index measured the predictive ability as the agreement between the predicted and observed times in any pair of randomly selected patients. The range of C-index in a model can range between 0.5 (represents random chance) and 1.0 (represents a perfectly discriminating model). Calibration, which compared the predicted survival with actual survival, was evaluated using the Hosmer-Lemeshow test.

The difference between two estimates derived from the prediction models was the net survival benefit of adjuvant TACE, which could be interpreted as an expected survival difference when a patient underwent adjuvant TACE or not. Based on given input parameters, an Internet browser-based software tool was programmed in JavaScript, which provided an individualized prediction of the net survival benefit associated with adjuvant TACE, including the expected net survival time, as well as the increased 3- and 5-year survival probabilities. A value of *P* < 0.05 was considered statistically significant in this study. Data were analyzed using the SPSS software version 25.0 (SPSS, Chicago, IL, USA) and R 3.5.3 (<http://www.r-project.org/>), with package including “rms”, “foreign”, “survival”, “source (stdca.R)”, and “survminer”.
