# Supplementary figures and images for: Development and validation of a novel online calculator for estimating survival benefit of adjuvant transcatheter arterial chemoembolization in patients undergoing surgery for hepatocellular carcinoma
Source: J Hematol Oncol. 2021 Oct 12;14:165. doi: 10.1186/s13045-021-01180-5 (PMC8507320; doi:10.1186/s13045-021-01180-5)

**Figure S1.** Flow chart of patient inclusion.


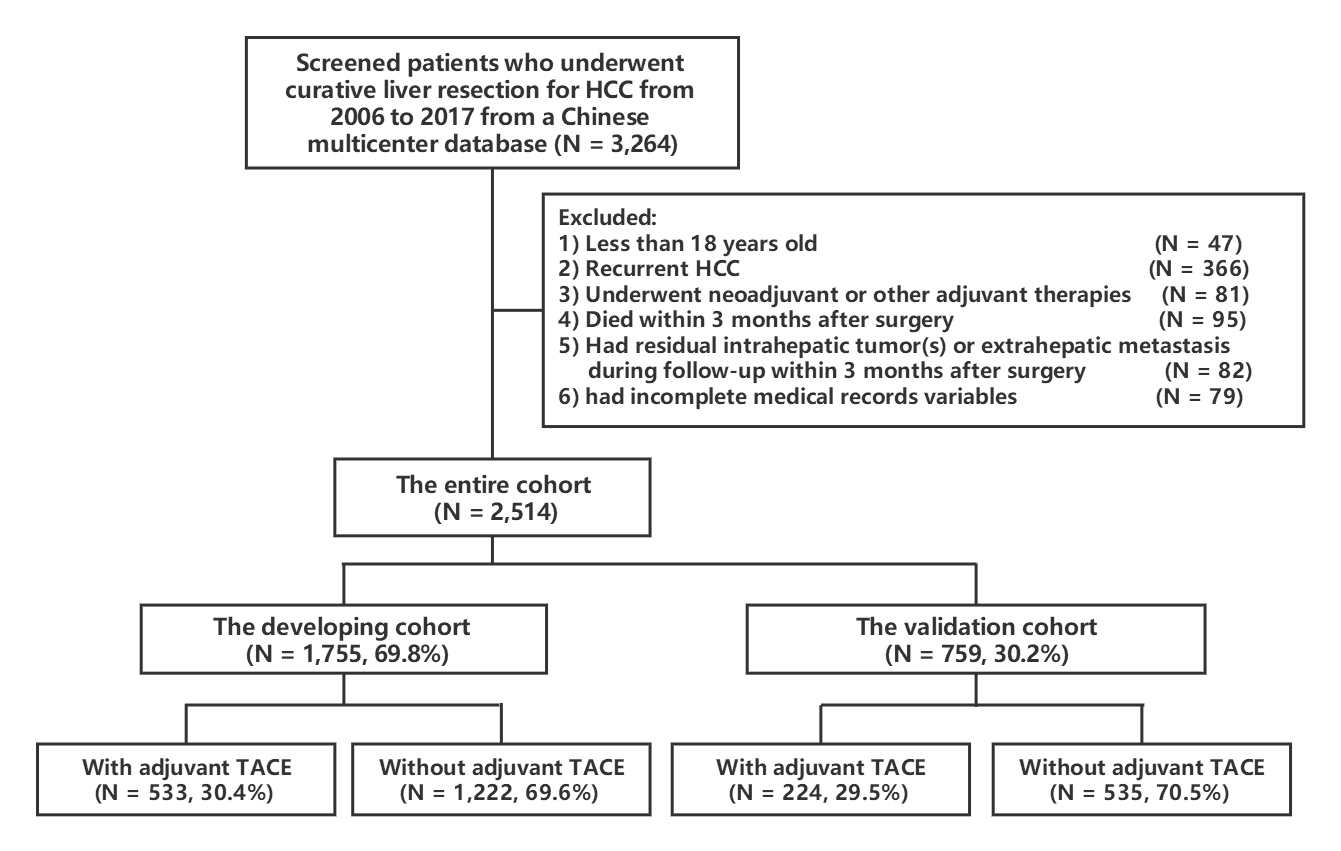

Supplement: Supplementary file 2 — Additional file 2: Figure S1. Flow chart of patient inclusion. [file 13045_2021_1180_MOESM2_ESM.docx]
