# Supplementary material for: Development and validation of a novel online calculator for estimating survival benefit of adjuvant transcatheter arterial chemoembolization in patients undergoing surgery for hepatocellular carcinoma
Source: J Hematol Oncol. 2021 Oct 12;14:165. doi: 10.1186/s13045-021-01180-5 (PMC8507320; doi:10.1186/s13045-021-01180-5)

Figure S2. (A) Kaplan-Meier curves of overall survival between patients in the development and validation cohorts (log-rank test, *P* = 0.594); (B) Kaplan-Meier curves of overall survival between patients with and without adjuvant TACE in the developing cohort (log-rank test, *P* < 0.001); and (C) Kaplan-Meier curves of overall survival between patients with and without adjuvant TACE in the validation cohort (log-rank test, *P* < 0.001). Number of patients at risk and censoring were list in the box at the bottom of each plot.


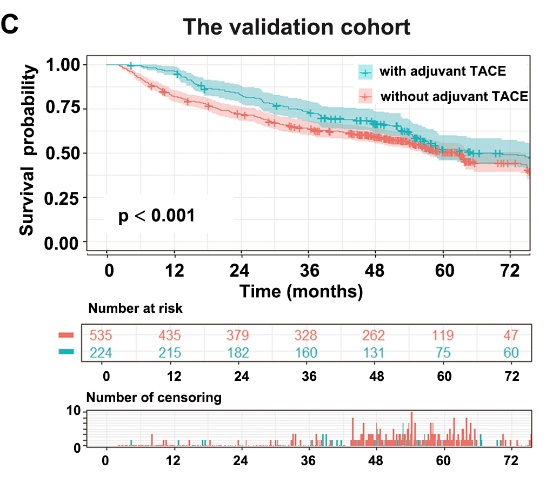

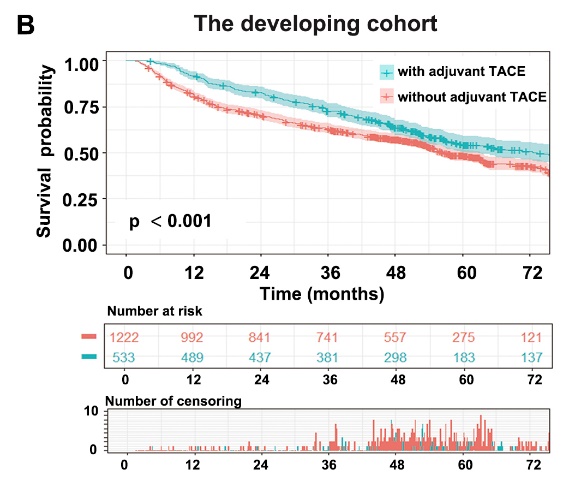

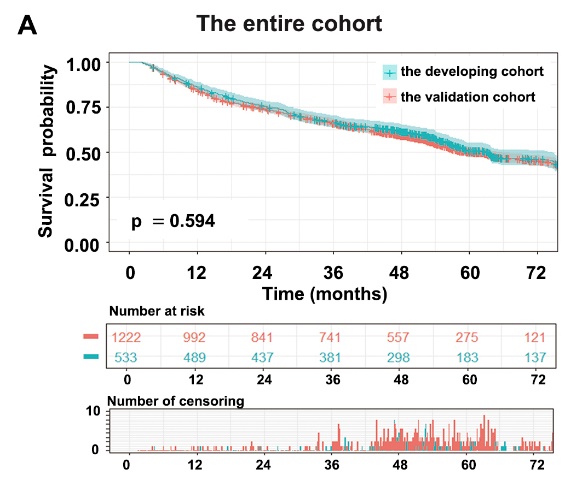

Supplement: Supplementary file 3 — Additional file 2: Figure S2. (A) Kaplan-Meier curves of overall survival between patients in the development and validation cohorts (log-rank test, P = 0.594); (B) Kaplan-Meier curves of overall survival between patients with and without adjuvant TACE in the developing cohort (log-rank test, P < 0.001); and (C) Kaplan-Meier curves of overall survival between patients with and without adjuvant TACE in the validation cohort (log-rank test, P < 0.001). Number of patients at risk and censoring were list in the box at the bottom of each plot. [file 13045_2021_1180_MOESM3_ESM.docx]
