# Supplementary material for: Development and validation of a novel online calculator for estimating survival benefit of adjuvant transcatheter arterial chemoembolization in patients undergoing surgery for hepatocellular carcinoma
Source: J Hematol Oncol. 2021 Oct 12;14:165. doi: 10.1186/s13045-021-01180-5 (PMC8507320; doi:10.1186/s13045-021-01180-5)
Supplement: Supplementary file 4 — Additional file 3: Table S1. Univariable and multivariable Cox-regression analyses of predicting overall survival for patients with resected hepatocellular carcinoma who underwent adjuvant TACE in the developing cohort. [file 13045_2021_1180_MOESM4_ESM.docx]

**Table S1.** Univariable and multivariable Cox-regression analyses of predicting overall survival for patients with resected hepatocellular carcinoma who underwent adjuvant TACE in the developing cohort.

| **Variables** | **HR Comparison** | **UV HR (95% CI)** | **UV *P*** | **MV HR (95% CI)** | **MV *P*** |
| --- | --- | --- | --- | --- | --- |
| Sex | Male vs. Female | 1.024 (0.806-1.302) | 0.844 |  |  |
| Age | > 60 vs. ≤ 60 year | 1.017 (0.843-1.227) | 0.862 |  |  |
| Co-morbid illness | Yes vs. No | 1.055(0.867-1.282) | 0.596 |  |  |
| PS score | 1-2 vs. 0 | 1.128 (0.954-1.334) | 0.158 |  |  |
| ASA score | > 2 vs. ≤ 2 | 1.033 (0.679-1.572) | 0.880 |  |  |
| Etiology of liver disease | HBV vs. non-HBV | 1.131 (0.580-2.203) | 0.718 |  |  |
| Cirrhosis | Yes vs. No | 1.174 (0.896-1.539) | 0.245 |  |  |
| [Portal](javascript:;) [hypertension](javascript:;) | Yes vs. No | 1.603 (1.349-1.905) | < 0.001 | 1.480 (1.243-1.762) | 0.001 |
| Child-Pugh grade | B vs. A | 2.581 (2.101-3.170) | < 0.001 | 1.861 (1.510-2.294) | < 0.001 |
| Preoperative ALT level | > 40 vs. ≤ 40 U/L | 1.145 (0.093-1.340) | 0.093 |  |  |
| Preoperative AST level | > 40 vs. ≤ 40 U/L | 1.073 (0.917-1.256) | 0.379 |  |  |
| Preoperative AFP level | > 400 vs. ≤ 400 ug/L | 2.279 (1.952-2.662) | < 0.001 | 1.627 (1.382-1.915) | 0.001 |
| Maximum tumor size | < 5.0 cm | 1.000 Reference |  | 1.000 Reference |  |
|  | 5.0~9.9 cm | 1.844 (1.545-2.202) | < 0.001 | 1.448 (1.207-1.738) | 0.002 |
|  | ≥ 10.0 cm | 3.551 (2.888-4.365) | < 0.001 | 1.799 (1.436-2.252) | < 0.001 |
| Tumor number | 1 | 1.000 Reference |  | 1.000 Reference |  |
|  | 2 | 2.676 (2.126-3.369) | < 0.001 | 1.885 (1.485-2.392) | 0.029 |
|  | ≥ 3 | 3.382 (2.767-4.134) | < 0.001 | 2.268 (1.840-2.795) | 0.001 |
| Macrovascular invasion | Yes vs. No | 6.640 (5.288-8.337) | < 0.001 | 3.405 (2.684-4.318) | < 0.001 |
| Intraoperative blood loss | > 600 vs. ≤ 600 ml | 1.753 (1.333-2.304) | < 0.001 | 1.154 (0.813-1.638) | 0.424 |
| Intraoperative blood transfusion | Yes vs. No | 1.560 (1.208-2.016) | 0.001 | 1.052 (0.772-1.434) | 0.749 |
| Operation time | > 180 vs. ≤ 180 min | 1.294 (0.936-1.788) | 0.119 |  |  |
| Non-anatomical resection | Yes vs. No | 1.026 (0.784-1.343) | 0.851 |  |  |
| Major hepatectomy | Major vs. Minor | 2.229 (1.734-2.866) | < 0.001 | 1.192 (0.832-1.709) | 0.338 |
| Microvascular invasion | Yes vs. No | 2.303 (1.962-2.703) | < 0.001 | 1.644 (1.390-1.945) | < 0.001 |
| Poor tumor differentiation | Yes vs. No | 1.700 (1.196-2.417) | 0.003 | 1.031 (0.706-1.504) | 0.876 |
| Incomplete tumor encapsulation | Yes vs. No | 2.871 (2.154-3.827) | < 0.001 | 1.074 (0.808-1.428) | 0.624 |
| Resection margin | < 1.0 vs. ≥ 1.0 cm | 1.683 (1.409-2.009) | < 0.001 | 1.379 (1.150-1.653) | 0.001 |

AFP, alpha-fetoprotein; ALT, alanine aminotransferase; ASA, American Society of Anesthesiologists; AST, [aspartate](javascript:;) [transaminase](javascript:;); CI, confidence interval; HBV, hepatitis B virus; HR, hazard ratio; MV, multivariable; PS, performance status; TACE, transcatheter arterial chemoembolization, UV, univariable.
