# Supplementary material for: Development and validation of a novel online calculator for estimating survival benefit of adjuvant transcatheter arterial chemoembolization in patients undergoing surgery for hepatocellular carcinoma
Source: J Hematol Oncol. 2021 Oct 12;14:165. doi: 10.1186/s13045-021-01180-5 (PMC8507320; doi:10.1186/s13045-021-01180-5)
Supplement: Supplementary file 5 — Additional file 2: Table S2. Univariable and multivariable Cox-regression analyses of predicting overall survival for patients with resected hepatocellular carcinoma who did not undergo adjuvant TACE in the developing cohort. [file 13045_2021_1180_MOESM5_ESM.docx]

**Table S2.** Univariable and multivariable Cox-regression analyses of predicting overall survival for patients with resected hepatocellular carcinoma who did not undergo adjuvant TACE in the developing cohort.

| **Variables** | **HR Comparison** | **UV HR (95% CI)** | **UV *P*** | **MV HR (95% CI)** | **MV *P*** |
| --- | --- | --- | --- | --- | --- |
| Sex | Male vs. Female | 0.989 (0.677-1.429) | 0.929 |  |  |
| Age | > 60 vs. ≤ 60 year | 1.051 (0.780-1.417) | 0.742 |  |  |
| Co-morbid illness | Yes vs. No | 1.006 (0.760-1.333) | 0.965 |  |  |
| PS score | 1-2 vs. 0 | 1.227 (1.064-1.532) | 0.009 | 1.023 (0.845-1.238) | 0.817 |
| ASA score | > 2 vs. ≤ 2 | 1.350 (1.039-1.674) | 0.006 | 1.032 (0.824-1.291) | 0.786 |
| Etiology of liver disease | HBV vs. non-HBV | 1.185 (0.682-2.061) | 0.547 |  |  |
| Cirrhosis | Yes vs. No | 1.084 (0.876-1.341) | 0.457 |  |  |
| [Portal](javascript:;) [hypertension](javascript:;) | Yes vs. No | 2.419 (2.005-2.918) | < 0.001 | 1.675 (1.263-2.220) | 0.001 |
| Child-Pugh grade | B vs. A | 2.474 (1.919-3.190) | < 0.001 | 1.815 (1.244-2.647) | 0.002 |
| Preoperative ALT level | > 40 vs. ≤ 40 U/L | 1.073 (0.848-1.356) | 0.558 |  |  |
| Preoperative AST level | > 40 vs. ≤ 40 U/L | 1.068 (0.847-1.347) | 0.576 |  |  |
| Preoperative AFP level | > 400 vs. ≤ 400 ug/L | 2.807 (2.367-3.329) | < 0.001 | 1.828 (1.391-2.403) | < 0.001 |
| Maximum tumor size | < 5.0 cm | 1.000 Reference |  | 1.000 Reference |  |
|  | 5.0~9.9 cm | 1.750 (1.439-2.129) | < 0.001 | 1.390 (1.001-1.928) | 0.049 |
|  | ≥ 10.0 cm | 3.891 (3.094-4.893) | < 0.001 | 2.053 (1.398-3.015) | < 0.001 |
| Tumor number | 1 | 1.000 Reference |  | 1.000 Reference |  |
|  | 2 | 3.605 (2.762-4.707) | < 0.001 | 1.501 (1.076-2.095) | 0.017 |
|  | ≥ 3 | 5.435 (4.328-6.825) | < 0.001 | 2.034 (1.505-2.749) | < 0.001 |
| Macrovascular invasion | Yes vs. No | 8.796 (6.892-11.224) | < 0.001 | 2.629 (1.839-3.757) | < 0.001 |
| Intraoperative blood loss | > 600 vs. ≤ 600 ml | 1.526 (1.249-1.863) | 0.001 | 0.993 (0.774-1.274) | 0.956 |
| Intraoperative blood transfusion | Yes vs. No | 1.392 (1.159-1.672) | < 0.001 | 1.036 (0.829-1.295) | 0.757 |
| Operation time | > 180 vs. ≤ 180 min | 1.102 (0.868-1.399) | 0.427 |  |  |
| Non-anatomical resection | Yes vs. No | 1.132 (0.934-1.371) | 0.206 |  |  |
| Major hepatectomy | Major vs. Minor | 1.504 (1.247-1.815) | < 0.001 | 1.000 (0.818-1.223) | 0.999 |
| Microvascular invasion | Yes vs. No | 2.326 (1.952-2.773) | < 0.001 | 1.474 (1.099-1.977) | 0.010 |
| Poor tumor differentiation | Yes vs. No | 1.110 (0.906-1.360) | 0.312 |  |  |
| Incomplete tumor encapsulation | Yes vs. No | 2.196 (1.887-2.556) | < 0.001 | 1.035 (0.839-1.278) | 0.746 |
| Resection margin | < 1.0 vs. ≥ 1.0 cm | 2.624 (2.127-3.238) | < 0.001 | 1.489 (1.170-1.895) | 0.001 |

AFP, alpha-fetoprotein; ALT, alanine aminotransferase; ASA, American Society of Anesthesiologists; AST, [aspartate](javascript:;) [transaminase](javascript:;); CI, confidence interval; HBV, hepatitis B virus; HR, hazard ratio; MV, multivariable; PS, performance status; TACE, transcatheter arterial chemoembolization, UV, univariable.
