# Supplementary material for: Development and validation of a novel online calculator for estimating survival benefit of adjuvant transcatheter arterial chemoembolization in patients undergoing surgery for hepatocellular carcinoma
Source: J Hematol Oncol. 2021 Oct 12;14:165. doi: 10.1186/s13045-021-01180-5 (PMC8507320; doi:10.1186/s13045-021-01180-5)
Supplement: Supplementary file 6 — Additional file 2: Table S3. The corresponding score and the formula of our nomogram models. [file 13045_2021_1180_MOESM6_ESM.docx]

**Table S3.** The corresponding score and the formula of our nomogram models.

| **Variables** | **Categories** | **Points (for patients with adjuvant TACE)** | **Points (for patients without adjuvant TACE)** |
| --- | --- | --- | --- |
| Portal hypertension | No | 0 | 0 |
|  | Yes | 51.94777 | 31.19859 |
| Child-Pugh grade | A | 0 | 0 |
|  | B | 60.05807 | 51.15008 |
| Preoperative AFP level | ≤ 400 ug/L | 0 | 0 |
|  | > 400 ug/L | 60.30426 | 39.33928 |
| Maximum tumor size | < 5.0 cm | 0 | 0 |
|  | 5.0 ~ 9.9 cm | 35.32567 | 25.82731 |
|  | ≥ 10.0 cm | 70.65135 | 51.65426 |
| Tumor number | 1 | 0 | 0 |
|  | 2 | 31.78122 | 35.10249 |
|  | 3 | 63.56243 | 70.40299 |
| Macrovascular invasion | No | 0 | 0 |
|  | Yes | 100 | 100 |
| Microvascular invasion | No | 0 | 0 |
|  | Yes | 39.30759 | 41.68323 |
| Resection margin | ≥ 1.0 cm | 0 | 0 |
|  | < 1.0 cm | 40.18231 | 25.85738 |
| **Points** = Portal hypertension + Child-Pugh + Preoperative AFP level + Maximum tumor size + Tumor number + Macrovascular invasion + Microvascular invasion + Resection margin. | | | |
| **For patients with adjuvant TACE:**  1) Expected survival time = 1.977e^-06^×Points^3^ + -0.0011054×Points^2^ + -0.084758999×Points + 93.891954078  2) 3-years survival probability = 1.9 e^-08^×Points^3^ + -1.6233 e^-05^×Points^2^ + 0.001217872×Points + 0.874181252  3) 5-years survival probability = 3.5 e^-08^×Points^3^ + -1.8669 e^-05^×Points^2^ + -0.000184557×Points + 0.82509684 | | | |
| **For patients without adjuvant TACE:**  1) Expected survival time = 7.594 e^-06^×Points^3^+-0.002434342×Points^2^ + -0.225062883×Points + 99.651076045  2) 3-years survival probability =3.7e^-08^×Points^3^+-2.0361 e^-05^×Points^2^+ -0.000396771×Points + 0.896251275  3) 5-years survival probability = 6.7e^-08^×Points^3^+-2.4271 e^-05^×Points^2^+ -0.001407053×Points + 0.806544868 | | | |
